# The Economic Impact of Eradicating Peste des Petits Ruminants: A Benefit-Cost Analysis

**DOI:** 10.1371/journal.pone.0149982

**Published:** 2016-02-22

**Authors:** Bryony A. Jones, Karl M. Rich, Jeffrey C. Mariner, John Anderson, Martyn Jeggo, Sam Thevasagayam, Yi Cai, Andrew R. Peters, Peter Roeder

**Affiliations:** 1 Production and Population Health Department, Royal Veterinary College, University of London, Hatfield, United Kingdom; 2 Lab 863 Limited, Edgware, United Kingdom, and Norwegian Institute of International Affairs, Oslo, Norway; 3 Tufts Cummings School of Veterinary Medicine, North Grafton, Massachusetts, United States of America; 4 The Nelson Mandela African Institute of Science and Technology, Arusha, Tanzania; 5 Geelong Centre for Emerging Infectious Disease, Medical Faculty, Deakin University, Geelong, Australia; 6 Bill and Melinda Gates Foundation, Seattle, Washington, United States of America; 7 Scotland’s Rural College, Edinburgh, United Kingdom; 8 Taurus Animal Health, Headley Down, Hampshire, United Kingdom; Indian Institute of Science, INDIA

## Abstract

Peste des petits ruminants (PPR) is an important cause of mortality and production loss among sheep and goats in the developing world. Despite control efforts in a number of countries, it has continued to spread across Africa and Asia, placing an increasing burden on the livelihoods of livestock keepers and on veterinary resources in affected countries. Given the similarities between PPR and rinderpest, and the lessons learned from the successful global eradication of rinderpest, the eradication of PPR seems appealing, both eliminating an important disease and improving the livelihoods of the poor in developing countries. We conducted a benefit-cost analysis to examine the economic returns from a proposed programme for the global eradication of PPR. Based on our knowledge and experience, we developed the eradication strategy and estimated its costs. The benefits of the programme were determined from (i) the averted mortality costs, based on an analysis of the literature, (ii) the downstream impact of reduced mortality using a social accounting matrix, and (iii) the avoided control costs based on current levels of vaccination. The results of the benefit-cost analysis suggest strong economic returns from PPR eradication. Based on a 15-year programme with total discounted costs of US$2.26 billion, we estimate discounted benefits of US$76.5 billion, yielding a net benefit of US$74.2 billion. This suggests a benefit cost ratio of 33.8, and an internal rate of return (IRR) of 199%. As PPR mortality rates are highly variable in different populations, we conducted a sensitivity analysis based on lower and higher mortality scenarios. All the scenarios examined indicate that investment in PPR eradication would be highly beneficial economically. Furthermore, removing one of the major constraints to small ruminant production would be of considerable benefit to many of the most vulnerable communities in Africa and Asia.

## Introduction

Peste des petits ruminants (PPR) is an acute, contagious, and frequently fatal disease of sheep and goats, caused by a morbillivirus related to the viruses that cause rinderpest in cattle, measles in humans, and distemper in dogs [1]. After the successful global eradication of rinderpest in 2011, there were calls for the progressive control or eradication of PPR at regional or global level [2–4]. In 2011 the World Organisation for Animal Health (OIE) and the United Nations Food and Agriculture Organization (FAO) started to discuss the possibility of PPR progressive control leading to eradication and then formed a PPR working group. A number of countries have started national PPR control programmes, such as the Kingdom of Saudi Arabia, India, Pakistan and China. In March 2015, OIE and FAO officially launched a new programme to eradicate PPR by 2030 (http://www.oie.int/eng/ppr2015/background.html) and presented a global control and eradication strategy [5].

PPR disease is characterised by fever, ocular and nasal discharges, oral erosions, bronchopneumonia and diarrhoea [6]. The severity of clinical signs, the morbidity rate, and the case fatality rate can vary depending on the virulence of the virus strain, the species and breed of the host, concurrent infection, and previous exposure of the population to PPR virus (PPRV) [1]. Many wild artiodactyl species are also susceptible to PPR disease [7] but there is currently no evidence to suggest that disease is maintained in these populations without concurrent infection in local sheep or goats. PPR occurs in most parts of Africa, Turkey, the Middle East, and parts of Central, South and East Asia. It has extended its geographical range considerably in the last decade, emerging in northern Africa, southern Africa and China [1, 8, 9]. When introduced to a naïve population, morbidity and mortality can reach almost 100%, causing a major shock to livestock keeper livelihoods and to small ruminant trade. Unless animal health services respond rapidly with effective control measures, the disease will become endemic, with a long-term negative impact on small ruminant productivity, especially for poorer and more marginalised livestock-keeping households.

Like rinderpest virus, PPR virus has many of the characteristics of an eradicable disease, as described by Miller *et al*.[10]. PPR vaccines currently in use are able to induce protective immunity against all known serotypes; immunity is lifelong, whether due to natural infection or vaccination; infection is transmitted primarily by direct contact and the virus does not persist in the environment; infected animals are infectious for a short period of time and there is no carrier state; while a number of different wildlife ungulate species can be infected, there is no evidence to indicate that wildlife populations play an important role in virus maintenance; an effective, robust, safe and affordable vaccine is available; a thermo-stable vaccine has been developed; and sensitive and specific diagnostic tests are available. Nevertheless there are some important differences between PPR and rinderpest that need to be considered. Goats and sheep are more numerous and reproduce more rapidly than cattle, which creates a much greater challenge for the vaccination strategy. Sheep and goats have a different socio-economic role compared to cattle: their value per head is lower, with an associated lower investment per head on health care, in spite of playing an important role in food security and livelihoods, utilising marginal grazing unsuitable for cattle or for crop production.

Vaccination is the main tool for controlling and eradicating PPR. Since the main route of transmission is by direct contact, movement control is also effective but is difficult to implement in many of the infected countries where extensive and mobile production systems are common. To date control strategies have been mainly based on annual national mass vaccination campaigns and/or focal vaccination in response to overt outbreaks. Theoretically, mass annual vaccination is an effective control measure, but in practice is difficult to achieve and is costly. This was the experience with mass vaccination for rinderpest in cattle but is an even greater challenge for sheep and goats because their population is larger with a higher turnover. Moreover, as disease incidence decreases after vaccination, it becomes even more difficult to justify and maintain high vaccination coverage, which leads to sporadic outbreaks, trapping the country into an open-ended annual vaccination programme. Whilst such vaccination campaigns can reduce the socio-economic impact of PPR, mathematical transmission models of the closely-related rinderpest virus indicate that sub-optimal vaccination coverage is likely to favour virus persistence [11]. A more effective time-bound strategy is therefore required which will achieve eradication and avoid the need for long-term costly control programmes.

Given that PPR has already been targeted by FAO and OIE as a high priority disease for global eradication, we present here a benefit-cost analysis of a proposed global strategy for the eradication of PPR, to support decision-making by international organisations and donors, as well as regional and national-level stakeholders, on whether a PPR eradication programme would be economically beneficial.

## Methods

### Development of Eradication Strategy

The authors developed a strategy for global PPR eradication through two 2-day meetings and email correspondence during 2013. Collectively, the authors have expertise in the following fields; animal health economics, veterinary epidemiology, livestock disease surveillance and control, laboratory diagnostics, research, and veterinary vaccines. The authors drew on their own knowledge and experience of rinderpest eradication and other trans-boundary disease control programmes including PPR, and reviewed the relevant literature. After agreeing on an overall strategy, the authors formed sub-groups to prepare specific parts of the strategy, i.e. diagnostics, research, vaccination, surveillance, economics, and governance, which were then reviewed and discussed by the whole group before finalising. The aim was to prepare a global framework of principles and approaches to achieve PPR eradication. Detailed planning for each region and country would be one of the activities of the strategy.

**Fig 1:**
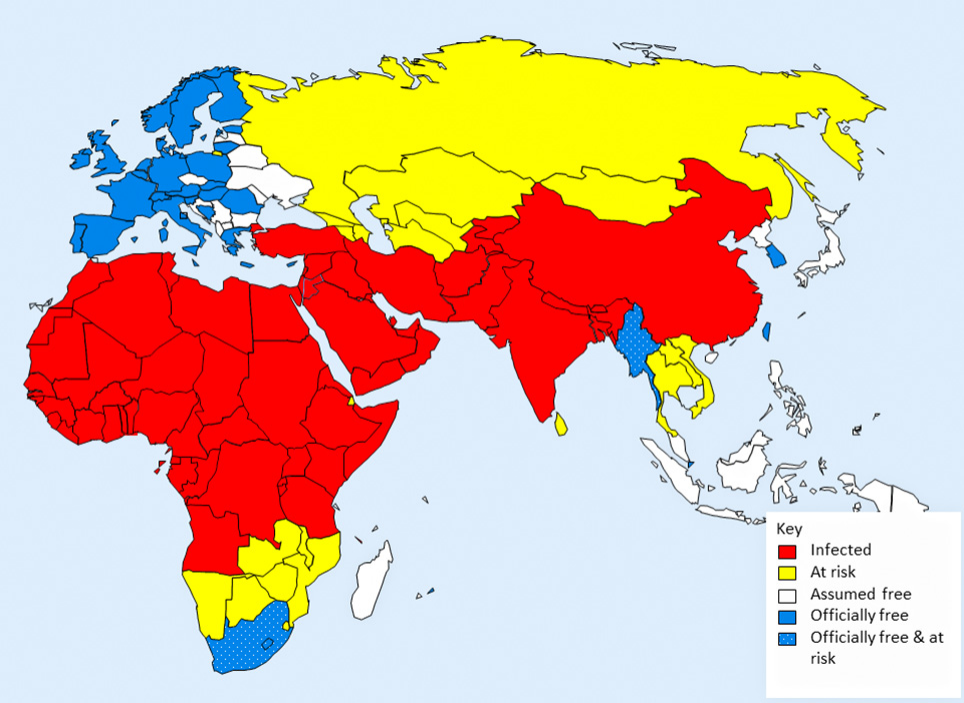
Spatial distribution of peste des petits ruminants. Based on data available up to 2014.

The strategy focussed on 65 countries that were known, or strongly suspected, to be infected, based on data available in OIE World Animal Health Information Database (WAHID) (http://www.oie.int/wahis_2/public/wahid.php/Wahidhome/Home), published articles, and the authors’ direct country experience. An additional 20 countries, adjacent to infected countries, were considered to be at high risk of PPR introduction (Fig 1, S1 Table).

#### Estimation of eradication programme costs

Based on the proposed strategy, timeframe, and the number of infected and at-risk countries, a global programme budget was estimated using the following budget categories; global coordination, regional coordination, national coordination, animal health institutional development, epidemiology and surveillance, diagnostic laboratories, vaccination, training and research, socio-economics, and contingency and emergency response capacity. A 3% annual inflation rate was applied, and the budget was discounted at 5% to adjust to net present value in year one, 2014.

#### Estimation of programme benefits

We broke down the prospective benefits associated with PPR eradication into three categories. Firstly, we estimated the economic impact associated with PPR mortality of sheep and goats in affected countries. Secondly, we used this estimate of mortality loss as an input to compute downstream impacts associated with PPR, using a social accounting matrix (SAM) to guide the magnitude of these potential downstream impacts in related and unrelated sectors [12, 13]. Thirdly, we estimated the avoided costs associated with current vaccination programmes. The benefits were projected over a 100-year period, given that the benefits remain in perpetuity, and were discounted at 5% to adjust to net present value in year one, 2014.

#### Avoided loss due to PPR mortality

We defined avoided losses associated with PPR-related mortality as follows in Eq (1).

Avoided loss due to PPR mortality=small ruminant population of infected countries x annual PPR mortality rate x value per animal (1)

This required obtaining information on population sizes, prices, and PPR mortality rates. To estimate the size of the sheep and goat population in infected countries, we obtained data on sheep and goat populations from FAOSTAT for the most recent year available (2011) (http://faostat.fao.org/site/573/DesktopDefault.aspx?PageID=573-ancor).

Using FAOSTAT data from 2011 on the live weight price per kg of sheep and goat meat per country, and carcass weight per animal per country (http://faostat.fao.org/site/291/default.aspx), we estimated the value of an average small ruminant in each infected country. For countries for which data were not available, we applied average values from neighbouring countries.

To estimate the annual PPR mortality rate in the infected population, we reviewed the published literature describing PPR outbreaks. As part of a wider literature review, the search term “peste des petits ruminants” was used in CABDirect, ScienceDirect and PubMed. The titles and abstracts were reviewed to find papers describing confirmed PPR outbreaks, or field studies of PPR morbidity and mortality. For each study, the within-flock mortality was extracted and a summary estimate of within-flock mortality was obtained by calculating the median and upper and lower quartiles. To determine the annual mortality rate at the population level, we applied nine scenarios with different lengths of epidemic cycle (2, 3 and 5 years) and different proportions of the flocks infected during each epidemic cycle (50, 70 and 90%), to obtain median and upper and lower quartiles for the proportion of the population exposed annually. Applying the estimates of median, and upper and lower quartile within-flock mortality to the nine annual flock infection scenarios, we estimated a median annual mortality rate for infected countries, and upper and lower quartiles to represent low and high mortality scenarios.

In the first few years of the programme we assume that there will be only a slight reduction in PPR mortality and therefore initially there will be no avoided losses due to PPR mortality, but once targeted vaccination starts to be implemented there will be a progressive reduction in annual PPR mortality and the avoided losses due to PPR mortality will increase each year until PPR disease no longer occurs. To take into account the progressive reduction in PPR mortality, we assumed that, in comparison to year 0 (the level of PPR mortality before the programme starts), there would be a 10% decrease in mortality in year 3, 30% in year 4, 45% in year 5, 60% in year 6, 75% in year 7, 95% in year 8, 98% in year 9, and 100% from year 10 onwards. We calculated the value of avoided loss due to PPR mortality for each year of the programme by multiplying the estimated value of current losses due to PPR mortality by the proportion of mortality averted for each year of the programme.

#### Downstream impacts due to reduced PPR mortality

We employed a social accounting matrix to guide the magnitude of potential downstream impacts due to the value of reduced PPR mortality. SAMs are an expansion of national input-output tables that denote economy-wide transactions (receipts and expenditures) within a country or region for all major economic sectors, factors of production, and household groups [12, 14]. Unlike input-output tables, SAMs typically provide more specificity and disaggregation of sector accounts and household groups in particular, making them well suited for macroeconomic analyses of poverty and consumption trends, for instance.

Input-output tables can be used to detail the linkages between productive sectors in the economy and to assess how changes in final demand (or supply) influence each sector. One means of doing this is through the calculation of SAM multipliers. SAM multipliers specify the magnitude of a one-unit change in final demand which can come from a change in export demand, government spending, or private investment, on the economic output of different sectors or household income [14]. In the case of an animal disease, however, the shock to the economy is typically a supply shock rather than a demand shock. In this case, an alternative type of SAM multiplier can be calculated (a “mixed-multiplier”) in which certain sectors are considered as supply-constrained (typically agriculture or livestock) while others are demand-driven (typically industrial sectors). In this case, sector income changes either from an exogenous change in output in supply-constrained sectors or final demand in demand-driven ones [15, 16].

Unfortunately, most SAMs do not adequately disaggregate the livestock sector into different sub-sectors to be able to do this type of analysis for each country potentially affected by PPR (e.g., beef, poultry, sheep/goats, etc.); indeed, some SAMs combine the livestock sector within agriculture, making such analysis even more difficult. The Kenya SAM of Kiringai *et al*. [17] is the only SAM known to the authors that has a separate account for sheep and goats. We thus computed mixed multipliers based on this SAM and applied these globally to each of the affected countries. This is likely to overestimate downstream effects in some countries, but underestimate them in others. These multipliers were applied to the value of avoided mortality to compute the downstream economy-wide impacts of PPR eradication on national economic output.

#### Avoided cost of current control programmes

We defined the annual cost of current control programmes as follows in Eq (2).

Annual cost of current control programmes=vaccination cost per animal x proportion of population vaccinated per year x population in infected countries (2)

This required obtaining information on cost per animal vaccinated, number of animals vaccinated, and small ruminant populations in infected countries. The estimated cost of vaccination per animal was based on the cost per rinderpest vaccination delivered during the Pan African Rinderpest Campaign (PARC) programme [18], which included all recurrent and fixed costs of the vaccination campaigns.

Data available in OIE WAHID on PPR vaccinations appeared to underestimate the proportion of the sheep and goat population vaccinated per year in infected countries. For example India reported no PPR vaccination in 2012 but we are aware of mass vaccination programmes in the north of India and targeted vaccination in the south of India. Therefore, based on levels of coverage in countries that had reported vaccination data to OIE and country reports of vaccination activities in regional meetings, we made the assumption that, on average, PPR-infected countries vaccinate 15% of their sheep and goats annually, and applied this to the total sheep and goat population in infected countries, to calculate the total number of sheep and goats vaccinated per year. Applying the cost of vaccination delivery per animal we obtained an estimate of the avoided losses associated with current vaccination programmes.

## Results

### Eradication Strategy

**Fig 2:**
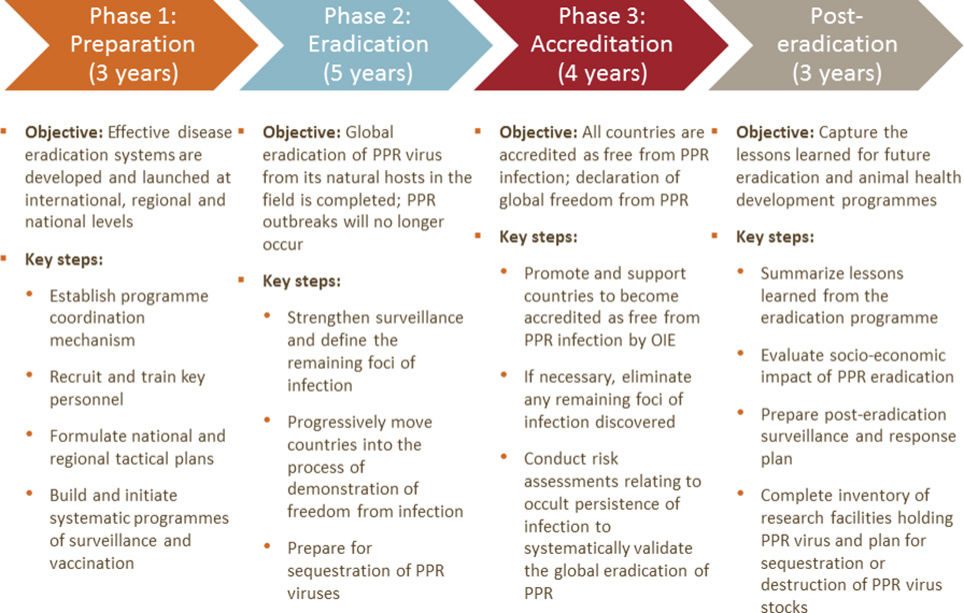
Outline of the proposed global strategy for PPR eradication.

**Fig 3:**
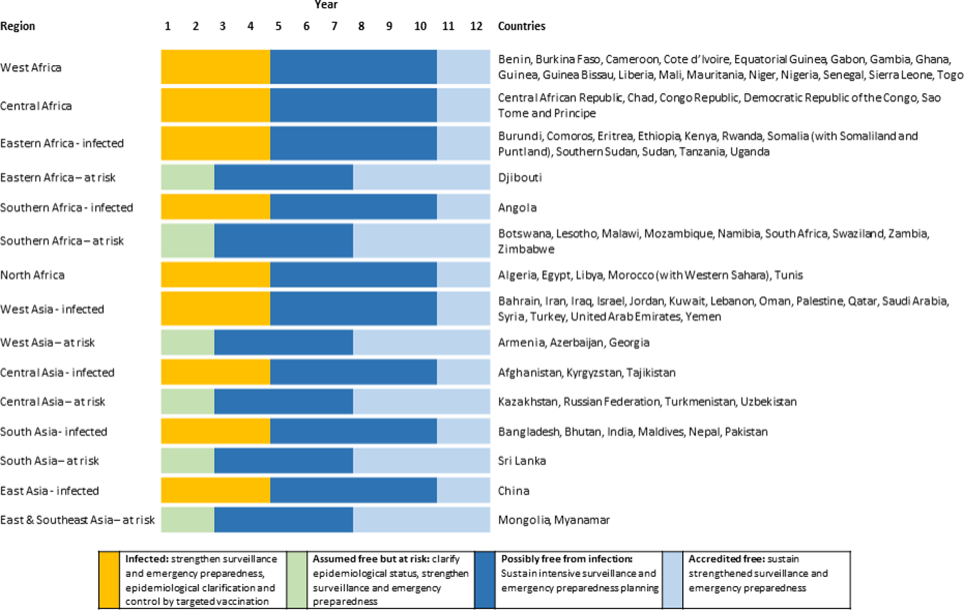
Initial disease status of countries and expected progress of eradication.

Here we present an overview of the proposed strategy and the details that are relevant for estimation of costs. The aim of the proposed global PPR eradication programme is to eradicate PPR within 12 years. The core pillars of the strategy are surveillance and field studies to understand the epidemiology of PPR and the high risk areas, followed by time-bound high-coverage vaccination targeted at these high risk areas to interrupt virus transmission. The programme is divided into a preparatory phase, an eradication phase, and an accreditation phase (Fig 2), although it is expected that some regions, and many countries, could progress more quickly depending on their initial disease status, existing PPR control activities, and existing animal health service delivery capacity (Fig 3). After achievement of eradication, there should be a post-eradication phase.

#### Phase I Preparation

The objective of this phase would be to develop and launch effective PPR eradication programmes at the international, regional and national levels. During this 3-year phase the focus would be on establishing coordination mechanisms at global, regional and national levels, training personnel in essential skills, undertaking the required research, collecting and analysing data on small ruminant populations and PPR epidemiology, developing national and regional plans or refining existing control programmes, strengthening surveillance systems and vaccination capacity, and commencing control activities in key populations. Establishing effective surveillance systems, supported by laboratory diagnostics, that use an appropriate combination of passive, active and risk-based methods would be a priority, to inform and guide targeted vaccination, and for early-warning systems in at-risk countries. Highly sensitive and specific diagnostic tests that are approved by OIE are already in use; competitive ELISA for antibody detection [19, 20] and immune-capture ELISA and Real-Time PCR for antigen detection [21, 22], as well as a rapid field diagnostic test for PPR antigen [23]. Epidemiological studies would be carried out to address knowledge gaps. Training would commence in this phase and continue into the second phase, to build critical core competencies in epidemiology and surveillance, livestock economic analysis, monitoring and evaluation, diagnostic services, and vaccine production and quality control.

#### Phase II Eradication

The objective of this phase would be to globally eliminate PPR virus from its natural hosts in the field so that by the end of this phase PPR outbreaks would no longer occur. During this 5-year phase, intensive targeted vaccination programmes underpinned by appropriate epidemiological studies would be carried out in infected countries to eradicate PPR virus. Under regional coordination, vaccination would be applied with the objective of interrupting virus transmission by identifying and targeting populations that are important for maintaining virus circulation, as indicated by surveillance and epidemiological studies, and should take no more than two years for most countries. Both infected and at-risk countries would need to conduct comprehensive surveillance to focus efforts on the elimination of infected foci, or to demonstrate absence of infection and move towards validation of freedom.

#### Phase III Accreditation.

The objective of this phase would be to systematically and scientifically validate the global eradication of PPR. During this 4-year phase, the focus would be on demonstrating the absence of PPR infection and preparing evidence for OIE accreditation of disease free status on a country-by-country basis. OIE requires a country to have ceased PPR vaccination for at least two years, and to provide sufficient epidemiological evidence to support recognition of disease freedom, which is provided as a dossier of disease-reporting data, active surveillance results and serological survey results.

#### Post-eradication phase

After achieving global freedom from PPR, there should be a 3-year post-eradication phase during which arrangements would be made for PPR virus sequestration, post-eradication surveillance and emergency response would be maintained, evaluations of impact and economic performance would be conducted, and lessons learned for future eradication and disease control programmes would be documented.

In some countries there will be important constraints such as limited animal health services, poor infrastructure, difficult environment or conflict. Based on experience with rinderpest eradication these will be addressed by building local capacity, using appropriate technology and methods for harsh environments and mobile communities, and supporting livestock keepers to carry out vaccination and surveillance in areas that are difficult to access [24].

The proposed strategy is intended to be flexible and should be adapted as implementation proceeds and lessons are learned. Learning and research should be integral parts of the programme to add to the existing knowledge base, and inform decision-making and strategy changes as the programme progresses.

We propose that the programme should be coordinated by an independent management team working under the guidance of a steering committee, the membership of which would be drawn from the main international organisations and donors concerned with livestock disease control, to encourage full participation and broad ownership across all stakeholders. A technical advisory group would guide strategy development and advise on technical issues. At regional and national levels, institutional arrangements would be tailored to fit the reality of local systems. Cross-border coordination would be essential because in most regions epidemiologically important populations span borders and involve multiple countries. This will be realised through strong regional coordination units that facilitate regional coordination meetings where countries share information and plan cross-border surveillance and vaccination activities so that epidemiological clusters of countries progress together towards PPR elimination.

At regional and national levels the strategy would be integrated with local small ruminant health initiatives to encourage participation. Where service delivery systems are inadequate, there would be investment in public and private animal health services that deliver PPR control through an integrated programme of basic animal health care, which also addresses several other high priority diseases impacting the local population. This would be especially relevant to under-served pastoralist areas with limited veterinary services.

Based on evidence from OIE WAHID, literature and experience, countries were assigned to one of four categories (Figs 1 and 3);

- infected; 65 countries with evidence, or strong suspicion, of PPRV infection;
- at-risk; 20 countries that are not yet considered to be PPR-infected but that neighbour infected countries and are likely to be infected in the near future if PPR is not controlled;
- officially free of infection; 48 countries are officially recognised by OIE to be free of PPR infection (by 2014);
- assumed free of infection; 75 countries/territories with no evidence of PPR infection and not at immediate risk.

#### Programme budget

Based on the proposed strategy, we estimated the human, operational and material resources that would be required to implement the programme for each year of each phase. We included an annual 3% adjustment factor to take inflation into account (Table 1, S2 Table). All figures given below are adjusted for inflation.

**Table 1:** Summary of programme budget by cost category and programme phase (US$ million).

Budget Category	Phase 1 (Years 1–3)	Phase 2 (Years 4–8)	Phase 3 (Years 9–12)	Post-eradication (Years 13–15)	Total
Global Coordination	7.5	14.1	12.9	3.5	38.0
Regional Coordination	9.4	17.6	16.1	4.3	47.5
National coordination	40.3	129.7	94.3	-	264.3
Animal health institutions	4.9	18.7	11.8	-	35.4
Epidemiology and surveillance	149.7	663.5	471.9	-	1,286.5
Diagnostic Laboratories	44.8	44.0	35.3	-	124.1
Vaccination	166.8	806.5	36.9	-	1,010.2
Training and research	62.9	35.2	6.5	1.3	105.9
Socio-economics	26.0	27.0	34.9	14.7	102.5
Contingency and emergency response capacity	13.0	27.3	21.1	5.0	66.4
Total Programme Cost	525.2	1,783.7	741.7	28.7	3,080.7

#### Global coordination (US$38 million)

The programme would be coordinated at a global level by an independent Executive Secretariat comprised of an 11-person team including a chairperson, technical advisor, regional advisors, monitoring officer, project manager and financial officer. The budget covers salaries and allowances, office costs, regional and national visits, steering committee meetings, technical advisory group meetings, and annual global coordination meetings.

#### Regional coordination (US$48 million)

There would be six regional coordination offices which would cover on average 14 infected or at-risk countries each. For each region, the budget covers salaries and allowances for two technical personnel, one regional coordination meeting per year, national visits, and administration and office costs.

#### National coordination (US$264 million)

A national programme coordination unit would be established in each of the 85 infected or at-risk countries, consisting of a national programme coordinator, epidemiologist, performance monitoring lead, socio-economist, laboratory specialist, laboratory technician, communications specialist, operations manager, and administrator. The budget covers the costs for the coordination unit salaries, two national coordination meetings per year, administration and office costs, and communication materials and activities. The duration and size of the national programmes would vary depending on the size and complexity of the small ruminant sector, the existing capacity of the animal health services, and the initial PPR status. We have budgeted for an average national programme timeframe of 6.5 years for the 85 countries (S3 and S4 Tables). The mean small ruminant population of infected countries is 22.5 million (median 7 million), and for at-risk countries is 9 million (median 4.5 million). Taking into account the fewer small ruminants and lower level of programme activities in at-risk countries, we have budgeted for 50% salary costs for these countries.

#### Animal health institutional development (US$35 million)

During the first year of its programme, each participating country would hold a workshop to discuss and find solutions to important weaknesses in animal health service delivery. Funds would be allocated to support strengthening of animal health service delivery based on national needs (an average of US$323,000 per country).

#### Epidemiology and surveillance (US$1.3 billion)

An initial activity in all countries would be the collation of existing epidemiological data, followed by epidemiological studies to address knowledge gaps, and ongoing data collection during the programme (US$569,000 per country). In the early stages of the programme a workshop would be held in each country to discuss and find solutions to any important weaknesses in the disease surveillance system. It is likely that use of digital technology could be a cost-effective way to address some weaknesses, so some budget has been included to support the introduction or strengthening of digital surveillance systems (US$41,000 per country). Field surveillance would be an important activity throughout the national programmes, involving field veterinarians and other animal health personnel, transport, cold chain, field equipment, sampling equipment and rapid diagnostic test kits. It is assumed that animal health personnel would spend on average 7% of their time per year on PPR surveillance. The cost is estimated to average US$15.9 million for 6 years of surveillance in an infected country, and US$4.0 million in an at-risk country (total cost = US$1.23 billion).

#### Diagnostic laboratories (US$124 million)

It is proposed that all participating countries should have a nationally accredited laboratory that can undertake PPR serology and basic virus identification. This would require a one-off cost of equipping 85 suitable laboratories (10 local, 69 national and 6 regional reference laboratories), and training of personnel. There would be recurrent costs associated with laboratory operation (supply of reagents, equipment replacement and upgrading) and national serum bank maintenance. We have budgeted for annual regional co-ordination meetings for the diagnostics network, a quality assurance programme, support for the World Reference Laboratory for Morbillivirus (WRLM), and the cost of field sample submission to Regional and World Reference Laboratories.

#### Vaccination (US$1.01 billion)

Under the proposed strategy, vaccination would only be conducted in infected countries. The design of individual national vaccination strategies would depend on the local epidemiological situation based on surveillance findings and field studies in the early stage of the programme, but should be intensive and targeted. Therefore the actual level and frequency of vaccination would be likely to vary widely between countries. However, for budgeting purposes, we have estimated that a country would vaccinate in 3 rounds with an average coverage of 50% per round. The cost per vaccine dose (one dose is required per animal) is assumed to be US$0.10 based on current commercial prices in Africa, giving a total vaccine cost of US$246.8 million. Delivery of the vaccine to the animals would involve field veterinarians and other animal health personnel, transport, cold chain, field equipment, and vaccination equipment. It is assumed that animal health personnel would spend on average 10% of their time for two years on vaccination. The estimated cost per dose delivered is US$0.3, giving a total delivery cost of US$740.5 million. We have also budgeted for initial validation of thermo-stable vaccine candidates in the first two years of the programme, as well as ongoing vaccine quality assurance.

#### Training and research (US$106 million)

To strengthen national epidemiology and economics capacity, country-level training courses (US$43.3 million) and graduate training (MSc, PhD–US$ 32.7 million) are budgeted. US$2.1 million has been allocated for vaccine production and quality assurance training, and US$8.07 million for diagnostics training. To support national coordination units, funds are allocated to support epidemiology and economics expert missions (US$9.0 million).

A research budget is allocated to support research in support of the eradication programme (US$10.7 million) to address epidemiology knowledge gaps, socio-economic research, and diagnostics research.

#### Socio-economics (US$103 million)

Socio-economics costs include national operational costs throughout the programme, to conduct research to identify incentives that drive stakeholder participation in disease control, implement programmes to test enhancements of animal health institutional performance, and to collect baseline data and then monitor the impact of the eradication programme on livelihoods and livestock sector development (US$85.7 million). Regional socio-economic personnel would support and advise on the monitoring and evaluation of impact by national programmes (US$16.7 million).

#### Contingency and emergency response capacity (US$66 million)

Six regional vaccine banks containing 500,000 doses of vaccine would be established together with regional and national emergency response funds.

From the above calculations, the total estimated undiscounted cost of the global eradication programme would be US$3.08 billion. It is likely that most of the in-country personnel costs, which comprise approximately one third of the total budget, would be covered by national budgets. This estimate represents the minimum expected national contribution because in practice the contribution from richer countries is likely to be much higher.

### Programme Benefits

#### Avoided losses due to PPR mortality

The sheep and goat population of the 65 infected countries is estimated to be 1.44 billion based on 2011 FAOSTAT data (S1 Table).

In infected countries, the price per kg of sheep meat per country ranged from US$0.51 to US$11.23, with a median of US$2.60. For goat meat the minimum price was US$0.59 per kg and maximum was US$9.53, with a median of US$2.74. Data were missing for 22 infected countries so the values for similar neighbouring countries were applied (S5 Table).

In infected countries, the median average carcass weight per sheep per country was 13.2 kg (range 7.0–40.0 kg), and for goats the median was 11.9 (range 7.0–29.0 kg). For countries with missing data, the average value for the respective region was applied (S5 Table).

From 18 peer-reviewed papers describing PPR outbreaks in 9 countries, the median mortality in an affected flock was 13.2% (range 0–63%, inter-quartile range 4.9–26.3%) (S6 Table). Applying 9 scenarios with varying length of epidemic cycle (2, 3 and 5 years) and proportion of population exposed (50, 70 and 90%) we obtained a median annual exposure of 23.3% of the small ruminant population (range 10.0–45.0%) (S7 Table). Applying the within flock mortality to the proportion of the sheep and goat population exposed annually, we estimated a median annual mortality rate of 2.6% per year for infected countries. We also took the 25^th^ and 75^th^ percentiles (1.4% and 4.7%, respectively) to represent minimum and maximum mortality scenarios. Applying these mortality rates to the total sheep and goat populations in the 65 infected countries, we estimate that there are 37.4 million PPR-associated sheep and goat deaths each year (minimum 20.2 million, maximum 67.7 million), with a most likely total value of US$1,475 million, that could be as low as US$794 million or as high as US$2.7 billion.

Applying proportional reductions in PPR-associated mortality starting at 10% in year 3 of the eradication programme and increasing to 100% by year 10, the total discounted benefits of avoided mortality based on a 5% discount rate and a 100-year time horizon, the most likely value was estimated to be US$23.1 billion, but could be as low as US$12.5 billion or as high as US$41.8 billion (Table 2, S8 Table).

**Table 2:** Discounted benefits and costs of PPR eradication under different scenarios (US$ million).

Item	Low mortality scenario (1.4%)	Most likely scenario (2.6%)	High mortality scenario (4.7%)
Programme costs (discounted @5%)	2,264	2,264	2,264
Benefits (discounted @5%)	41,905	76,490	135,887
Discounted benefits, mortality	12,451	23,123	41,799
Discounted benefits, avoided vaccination losses	2,034	2,034	2,034
Discounted benefits, own sector effects	10,210	18,961	34,275
Discounted benefits, linkage effects on output	17,431	32,372	58,519
Net benefit	39,641	74,226	139,591
Benefit Cost Ratio (BCR)	18.51	33.78	60.02
BCR, mortality effects only	5.50	10.21	18.46
BCR, mortality and vaccination	6.40	11.11	19.36
Internal Rate of Return (IRR)	104%	199%	219%

#### Downstream impacts due to reduced PPR mortality

Applying the mixed multipliers derived from the Kenyan SAM to each of the affected countries, we found that the own-mixed multiplier for the sheep and goat sector was 0.82 and the downstream multiplier was 1.4. The own-multiplier of 0.82 means that an increase of US$1 in the supply of sheep and goats would increase economic output for the sheep and goat sector by US$0.82. Similarly, an increase by US$1 in the supply of the sheep and goat sector would have downstream output effects in all other economic sectors (agriculture, industry, transportation, etc.) valued at US$1.4. Using these multipliers and the estimated value of avoided mortality we computed the downstream economy-wide impacts of PPR eradication on national economic output: own-sector discounted benefits in the most likely scenario were estimated at US$19.0 billion, while downstream impacts were estimated at US$32.4 billion (Table 2, S8 Table).

#### Avoided cost of current control programmes

Applying an estimated cost per animal vaccinated of US$0.55 and an average annual vaccination coverage of 15% to the estimated 1.44 billion small ruminants in infected countries, we estimate that a total of 216 million sheep and goats are currently vaccinated per year in infected countries, at an estimated total cost of US$119 million. During the programme these costs would be replaced by the programme costs, and after the end of the programme and the eradication of PPR these costs would no longer be required. They are therefore the avoided costs associated with current PPR vaccination activities (Table 2, S8 Table).

#### Benefit-cost analysis of the eradication programme

Table 2 summarizes the results of the benefit-cost analysis under the three different PPR mortality scenarios; low mortality 1.4%, median mortality 2.6% (or most likely scenario), and high mortality 4.7%. We find that the benefit-cost ratio (BCR) of the proposed eradication programme in the most likely scenario is 33.8. This ranges from 18.5 in the low mortality scenario to 60.0 in the high mortality scenario. These are likely to be conservative estimates of the relative benefit of the programme, as we have not estimated losses due to reduced milk production, weight loss, and abortion related to PPR infection or the downstream impact of those losses. If we look only at the effects from reduced mortality, the BCR in the most likely scenario is 10.2. If we consider just the avoided losses of mortality and vaccination, the BCR rises to 11.1 in the most likely scenario. The IRR, taking into account all prospective benefits, in the most likely scenario is 199%, ranging from 104% in the low mortality scenario to 219% in the high mortality scenario.

## Discussion

Our analysis presents a compelling economic argument for global PPR eradication. A discounted expenditure of US$2.3 billion over 15 years could provide discounted benefits of US$76.5 billion, giving a net benefit of US$74.2 billion, a benefit cost ratio of 33.8 and an IRR of 199%. PPR mortality rates are highly variable and therefore we have included lower and higher mortality scenarios to estimate upper and lower values for the economic analysis. All the scenarios indicate that investment in PPR eradication is highly beneficial. Our calculations take into account avoided losses due to PPR mortality but not the reduction in milk yield, weight loss or abortion in sheep and goats that become sick but then recover, and therefore the benefits are likely to be underestimated.

We consider the eradication of PPR to be a global public good and therefore it is reasonable to expect contributions from the international community to support this effort. However we estimate that at least one third of the programme costs would be contributed by the individual countries, such as field staff salaries, and use of existing infrastructure, equipment and vehicles. Wealthier countries are likely to be able to cover all or most of their own costs, or even contribute to their regional programme costs.

The 12-year time-frame would be challenging but we believe it to be feasible. Having a shorter time-frame would reduce programme costs and stimulate timely action, leading to rapid progress and results, which would help to maintain momentum and commitment of the participating countries and other stakeholders.

We have estimated the programme costs at global, regional and national levels. The national level estimates are based on a hypothetical “average” country and therefore the true costs could vary significantly. However, even if the total programme budget doubled in size the benefit-cost analysis would still be very favourable.

For the estimated losses due to PPR mortality we obtained data from peer-reviewed papers, but the number of studies that were suitable for inclusion and the countries represented were limited, and data on national annual PPR mortality were not available so we had to extrapolate from flock level to population level. It is possible that the true mortality is higher than we estimated, which would lead to even more favourable economic returns to eradication. We believe mortality is unlikely to be lower than the lower value that we applied. In addition to uncertainty and variability in mortality, there is uncertainty due to missing or incomplete data for some countries such as small ruminant population estimates, the value of small ruminants, and current PPR vaccination coverage. We have therefore had to make assumptions about missing data. The PPR status for some countries is uncertain due to limited surveillance so we have been conservative and assigned them to “at-risk” rather than “infected” status. The analysis was carried out during 2013–2014 and therefore the PPR situation may have subsequently changed for some countries. If additional data becomes available it is simple to incorporate them into our model and update the estimates. If there is a delay before the start of the PPR eradication programme, then it is possible that PPR might spread to one or more of the at-risk countries. Again it is very simple to adjust the model to incorporate these changes.

We have assumed that sheep and goats are the primary hosts for the PPR virus and that no other domestic or wild species plays a significant role in PPR virus maintenance. There have been several reports of PPR disease in wildlife in Asia [25–29] but not Africa. It is highly likely that this is a spill-over infection from domestic animals and that control of PPR in small ruminants would eliminate infection from wildlife, as occurred during rinderpest eradication. There is evidence of infection of other domestic species but clinical disease has so far only been confirmed once in camels [30] and once in domestic buffalo [31], so it is unlikely that any control measure would be required in other domestic species.

Tambi *et al*. [18] reviewed the costs of rinderpest vaccination in 10 countries in Africa during the PARC Programme. They found an average cost of 0.42 ECU per animal vaccinated with a range of 0.27 (Ethiopia) to 1.71 (Ivory Coast). The average equates to US$0.55 per animal vaccinated, considering the value of the ECU in the 1990s. Tambi *et al*. [18] speculated that the low costs in Ethiopia were due to the large numbers vaccinated leading to economies of scale. Given the larger population sizes and the fact that sheep and goats are easier to handle than cattle, costs may be somewhat less. During the course of our analysis, the management of selected veterinary services were asked to provide an estimate of the cost of vaccination. They provided figures lower than the average determined by Tambi *et al*. [18], which could been due to an incomplete accounting of costs. The figures provided by Tambi *et al*. [18] were used without adjustment as the most transparent data available for current cost of vaccination. However for the cost of vaccination within the programme budget we estimated the costs of field personnel, transport, cold chain, equipment and vaccine to be US$0.40 per dose, which, if we also include the cost of administration and management that is costed separately in our budget, then our figure is similar that of Tambi *et al*. [18].

There are been several studies of the economic impact of PPR at farm, village, district and national levels. In a study of three outbreaks in Punjab Province, Pakistan, Abubakar and Munir [32] estimated the loss per animal in the three affected herds to be US$33, of which US$11.8 (35%) was due to the cost of mortality, and the balance was due to the cost of treatment and veterinary services, increased labour, loss of body condition and reduced market value. Thombare and Sinha [33] estimated the loss per animal in six infected villages in Maharastra, India to be US$7.7, of which US$2.0 (25%) was due to the cost of mortality: other costs included cost of treatment and veterinary services, weight loss, abortion, reduced wool and hair quality and reduced market value. Opasina and Putt [34] monitored villages in Nigeria and found that loss per head ranged from US$3.8 to 14.6 due to mortality only. A study of the 2006–2008 PPR outbreak in Turkhana District of Kenya estimated the value of losses due to mortality, and reductions in meat and milk over the period to be US$2.4 million, which is US$8 per animal in the district [35]. The loss due to mortality alone is not reported. In a review of the economic impact of small ruminant diseases in Nigeria, Akerejola *et al*. [36] estimate losses of 30–60 million Naira per annum due to PPR mortality (approximately US$48–96 million, or US$1.6–3.2 per animal in Nigeria). There have been several widely differing estimates of the annual losses due to PPR in India; US$0.66 million or US$0.003 per head of population due to mortality, milk and weight loss, and increased feed, management and medicine inputs [37, 38], US$36 million or US$0.16 per head [39] of which 83% is due to mortality (US$0.15 per head) and the balance is due to production losses and indirect costs such as export restrictions, US$39 million or US$0.21 per head [40] (types of loss not specified), and US$1,779 million or US$7.7 per head, of which 36% or US$2.6 per head is due to mortality and the balance is due to milk, weight and wool loss, abortion, treatment and increased management costs [41]. Our estimated annual total loss of US$1,475 million (794–2,666 million) due to PPR mortality only for a population of 1,440 million small ruminants in infected countries, gives a loss per head of US$1.0 (0.6–1.9), which is similar to the lower estimates in the above studies which ranged from US$0.003 to 14.6 per head. Only three studies reported the relative proportion of losses due to mortality compared to other production losses. These varied from 25% to 83%, indicating that the total direct losses due to PPR disease could be up to four times higher than our estimate based on losses due to mortality only.

Awa *et al*. [42] developed an economic model using parameters from an intervention study in northern Cameroon of PPR vaccination and helminth control and obtained an estimated BCR of 3.0 for sheep and 2.3 for goats over a 5 year period based on avoided mortality and increased replacement. Stem [43] used a dynamic herd model to estimate the benefits of PPR vaccination over a 5-year period in Niger and obtained BCR of 12, which is very similar to our BCR estimate of 10.2 based on avoided mortality only.

The OIE/FAO report on global PPR control and eradication [5] gives an estimated annual global economic loss of US$1.2–1.7 billion due to mortality, production loss and control costs; we estimated the annual loss due to mortality and control costs to be in the range of US$0.9–2.8 billion. They estimate the cost of an eradication programme to be between US$7.6 and 9.1 billion, which is 2.5–3 times our estimated budget. Their planning involves a longer time-frame (15 years), more countries involved (70 infected, 50 at-risk), and annual mass vaccination campaigns, rather than a targeted vaccination strategy. Their budget does not include critical enabling elements such as the strengthening of animal health institutions and capacity building.

If we look only at the effects from reduced mortality, the BCR in our most likely scenario is 10.2, significantly higher than the sector level BCR calculated by Tambi *et al*[18] in the context of rinderpest control under the PARC programme (BCR ranged from 1.1–3.8). Felton and Ellis [44] computed a BCR of 2.48 and an IRR of 48% for rinderpest control in Nigeria, while Tambi et al [45] estimated BCRs of 3.68 to 5.08 for different vaccination and surveillance strategies leading to rinderpest eradication in Ethiopia over a 12-year timeframe. The lower BCRs for rinderpest compared to our estimate for PPR are in part due to our analysis of PPR eradication (no disease incidence) rather than PPR control (reduced level of disease incidence), and our projection of the benefits of eradication over a 100-year timeframe rather than restricting them to the timeframe of the programme.

Using a SAM for downstream effects, we estimate that own-sector discounted benefits in the most likely scenario would be US$19.0 billion, while downstream impacts would be US$32.4 billion, confirming previous analysis of Rich and Wanyoike [46] that noted downstream effects of animal diseases are often larger than effects at the farm-level. Similar results on downstream effects were found by Ekboir [47], Garner and Lack [48], and Perry *et al*. [49] in the context of FMD control in the United States, Australia, and Zimbabwe, respectively.

From a technical standpoint. It is worth noting that the SAM analysis does not consider second-round impacts on prices or structural changes in the economy. Such an analysis would require the use of a computable general equilibrium model, or CGE, that uses a SAM as its baseline (see Rich *et al*. [50] for an application applied to rinderpest). Nonetheless, a SAM provides us with a relatively simple way of deriving the first-round economy-wide impact of eradicating PPR, particularly since we are likely to have underestimated the livelihood impact associated with PPR eradication in our analysis.

## Conclusion

This detailed analysis of both the costs and the benefits that would occur using the authors’ proposed strategy for the eradication of PPR demonstrates a large benefit-cost ratio, providing a compelling argument for proceeding with the eradication of PPR. Whilst there are likely to be additional social and developmental benefits that are difficult to quantify, the financial benefits alone more than justify the required expenditure.

## Supporting Information

S1 Table Sheep and goat population and PPR status. (XLSX)

S2 Table PPR Eradication Budget (including 3% annual inflation). (XLSX)

S3 Table National budget for an average infected country. (XLSX)

S4 Table National budget for an average at-risk country. (XLSX)

S5 Table Estimation of losses due to PPR mortality. (XLSX)

S6 Table PPR within-flock mortality literature review. (XLSX)

S7 Table Estimating annual mortality rate in infected countries. (XLSX)

S8 Table Benefit-cost analysis. (XLSX)
